# Joint spatial modeling to identify shared patterns among chronic related potentially preventable hospitalizations

**DOI:** 10.1186/1471-2288-14-74

**Published:** 2014-06-04

**Authors:** Berta Ibañez-Beroiz, Julián Librero, Enrique Bernal-Delgado, Sandra García-Armesto, Silvia Villanueva-Ferragud, Salvador Peiró

**Affiliations:** 1 NavarraBiomed – Fundación Miguel Servet - Red de Investigación en Servicios de Salud en Enfermedades Crónicas (REDISSEC), C/Irunlarrea s/n 31008, Pamplona, Spain; 2 Centro Superior de Investigación en Salud Pública (CSISP-FISABIO) - Red de Investigación en Servicios de Salud en Enfermedades Crónicas (REDISSEC), Valencia, Spain; 3 Instituto Aragonés de Ciencias de la Salud. IIS Aragón - Red de Investigación en Servicios de Salud en Enfermedades Crónicas (REDISSEC), Zaragoza, Spain; 4 European Commission, DG HEALTH & CONSUMERS (SANCO), Health Technology and Science Policy Officer, Brussels, Belgium

**Keywords:** Potentially preventable hospitalizations, Small-area analysis, Bayes theorem, Geographic information systems

## Abstract

**Background:**

Rates of Potentially Preventable Hospitalizations (PPH) are used to evaluate access of territorially delimited populations to high quality ambulatory care. A common geographic pattern of several PPH would reflect the performance of healthcare providers. This study is aimed at modeling jointly the geographical variation in six chronic PPH conditions in one Spanish Autonomous Community for describing common and discrepant patterns, and to assess the relative weight of the common pattern on each condition.

**Methods:**

Data on the 39,970 PPH hospital admissions for diabetes short term complications, chronic obstructive pulmonary disease (COPD), congestive heart failure, dehydration, angina admission and adult asthma, between 2007 and 2009 were extracted from the Hospital Discharge Administrative Databases and assigned to one of the 240 Basic Health Zones. Rates and Standardized Hospitalization Ratios per geographic unit were estimated. The spatial analysis was carried out jointly for PPH conditions using Shared Component Models (SCM).

**Results:**

The component shared by the six PPH conditions explained about the 36% of the variability of each PPH condition, ranging from the 25.9 for dehydration to 58.7 for COPD. The geographical pattern found in the latent common component identifies territorial clusters with particularly high risk. The specific risk pattern that each isolated PPH does not share with the common pattern for all six conditions show many non-significant areas for most PPH, but with some exceptions.

**Conclusions:**

The geographical distribution of the risk of the PPH conditions is captured in a 36% by a unique latent pattern. The SCM modeling may be useful to evaluate healthcare system performance.

## Background

The Potentially Preventable Hospitalizations (PPH), also named Potentially Avoidable Hospitalizations, Ambulatory Care Sensitive Conditions or Prevention Quality Indicators (PQI), are hospital admissions –predominantly exacerbation of chronic conditions– that conceptually may be preventable with timely and appropriate outpatient care [1,2]. The PPH are defined geographically (districts, municipalities, areas served by a primary care team or by a hospital, regions, etc.) based on the location of the patient’s residence and, succinctly, PPH could be defined as hospitalization rates for definite conditions from residents of delimited geographical areas, regardless of whether the hospital is located inside or outside the patient's area [2]. PPH rely on hospital discharge data, but are not intended as measures of in-hospital quality. Rather, they were built as indirect measures of accessibility problems to high quality outpatient care [3].

PPH, mainly in its PQI form, have been adopted (and adapted) by different national and international organizations [4-6] and currently are a common instrument for the evaluation of health care with expanded uses [7,8]. In countries with health systems without universal coverage, the interpretation of PPH has been oriented towards the identification of gaps in insurance coverage, access to outpatient care and the volume of primary care supply [9-14]. In Europe, where insurance tends to be universal and primary care is extensively developed, interpretations have been directed towards the evaluation of the quality of ambulatory care, and often, specifically referred to the quality of the primary care level, disregarding issues related with the role of outpatient care provided by specialists, the hospital responsibility in the control of chronic patients (discharging patients more or less stable, deciding which patients are admitted based on their own admission policies, etc.) and, also, the critical importance of a proper coordination between the different levels of care [15].

In the Spanish National Health System context, with an extended network of healthcare centres structured in two levels (hospitals and specialized outpatient care, and primary care) and geographically ordered (Hospital Departments and Primary Care Basic Health Zones (BHZ)), it is assumed that PPH represent largely a problem of coordination in the necessary chronic diseases continuum of care, both between and within levels of care [15,16]. This lack of coordination can affect similarly the full range of preventable hospitalizations, or may affect differently to each of the clinical conditions comprising PPH. In the former case, a common geographic pattern of the full range of PPH would reflect the quality and performance of healthcare providers, and could help to identify territories that handle homogeneously -better or worse - the most common chronic conditions causing PPH. In the latter, specific maps for each PPH would point out idiosyncratic organizational factors operating in the management of a particular PPH. It is expected both features to be present in our context, but in which extent has not been studied yet.

From a methodological perspective, given that the full range of preventable hospitalizations may share some of the aforementioned features, the conjoint analysis of them can be recommendable to gain in statistical power and interpretation. Additionally, the geographically structured nature of the data requires specific spatial methods to obtain unbiased estimates. Under the disease mapping setting, the Shared Component Model (SCM) [17] is a method that allows both the joint analysis of several diseases and the incorporation of spatial correlation, and has also shown to lead to improved inference over separate analysis of each outcome [18-21].

The aim of this paper is to explore the underlying pattern shared by six chronic PPH conditions in healthcare geographic BHZ of the Valencia Community (Spain) using Bayesian joint modeling, and assuming that the geographic areas serve as surrogate for a mix of the epidemiologic and medical practice risk factors that underlie any spatial variation in the pattern of hospital admissions.

## Methods

### Design

Population based cross-sectional ecological study, using the “Basic Health Zones” as unit of analysis.

### Setting

The study was conducted in the Valencia Community, an Autonomous Region on the Mediterranean coast of Spain, with approximately five million inhabitants. Like all of the healthcare system in Spain during the period studied, [22,23] healthcare coverage is practically universal, being 97% of the population covered by the public Health Service of the Valencia region (the Valencia Health Agency, VHA). The VHA operates an extended network of hospitals (84% of the hospital beds in the region) and primary healthcare centres (PHC). Some key features of the healthcare system during the study period are the following: health care in this network is free of charge (except for drugs in non-retired people who have a 40% co-payment), hospital and primary care is supported by Regional Government budgets, and doctors and other healthcare workers, who enjoy a civil servant-like status, are paid basically by salary. In 2009, the VHA was organized in 23 Healthcare Areas (three of them managed by private companies through Public-Private Partnership agreements) and 240 Basic Health Zones. Healthcare Areas are geographical territories between 75,000 and 500,000 people (most of them between 150,000-250,000 people) served by one public hospital that provides inpatient and outpatient specialized care to the BHZs of its demarcation. BHZs are small geographical areas between 1,000 and 65,000 people (most of them between 10,000 and 25,000 people) commonly served by one Primary Healthcare Centre with a stable team of doctors, nurses and other healthcare workers. Due to these organizational characteristics (geographical planning, minimal accessibility barriers, and practical absence of economic incentives to providers), patients receive most of their primary care from the Primary Healthcare Center of the BHZ where they belong, and most of their specialized care, including outpatient consultations, from the hospital of the corresponding Healthcare Area.

### Sources of data

The Population Information System, called SIP, a record of the population covered by the VHA that assigns an identification number to each individual, supplied the population denominator. Among other information, this dataset provides the Healthcare Area, the BHZ and Primary Healthcare Centre to which each individual belongs. The numerator (PPH admissions), was built using anonymized data from the Minimum Basic Hospital Discharge Dataset of the VHA from 2007 to 2009. This database, similar to the US Uniform Hospital Discharge Dataset, provides clinical and sociodemographic information on all hospital discharges in the VHA, and, basically, it is a synopsis of the patient episode of care, including diagnoses and procedures coded according to the International Classification of Diseases 9^th^ revision Clinical Modification (ICD9CM). The VHA Hospital Discharge Dataset includes the patient’s BHZ of residence and, once anonymized, was transferred to the research team.

### Population

The study population consisted of all residents 15 years and over that were registered in the SIP register in the period 2007–2009. The data was aggregated by five-year age-sex groups and BHZ.

### Main outcome measure

Age-sex standardized rates by 100,000 person-years in each BHZ, of six chronic PPH: diabetes short-term complications, chronic obstructive pulmonary disease (COPD), congestive heart failure (CHF), dehydration, angina admission and adult asthma. All 2007 to 2009 hospital admissions -readmissions included- of patients aged 15 years and over with a main diagnosis one of these PPH were selected and aggregated by age-sex groups and BHZ. For PPH operative definitions, we used the criteria of the Spanish validation [24,25] of the US Agency for Healthcare Research and Quality (AHRQ) Prevention Quality Indicators [2]. This Spanish version is similar to the US version, but some ICD9CM codes were adapted to the codification patterns most common in Spain and are fully described in a previous work [25,26].

### Ethical aspects

This study, observational in design, uses retrospective anonymized non-identifiable and non-traceable data provided upon request by the Health Department of the Valencia Regional Government (not the similar and freely available MBDS from the Spanish Ministry of Health). The authors declare that the transfer of the data to the research team met the requirements of the provider and, according to the CIOMS-WHO International Ethical Guidelines for Epidemiological Studies [27] and the Spanish personal data protection [28] and patients rights’ laws, [29] did not require Ethics Committee approval.

### Statistical Analysis

First, age-standardized rates were obtained for each BHZ and each single condition, together with global PPH frequencies and global age-standardized PPH rates. Variability among rates was quantified using the Extremal Quotient, excluding areas outside the percentiles 5 and 95 (EQ5-95), and the Empirical Bayes statistic (EB). Standardized Hospitalization Ratios (SHR) were estimated for each condition using the ratio of observed-to-expected cases (o_ij_/e_ij_, being o_ij_ and e_ij_ the observed and expected number of cases for BHZ *i* and PPH condition *j*), and correlations among these were also assessed. The expected number of cases per BHZ unit for each PPH, estimated by applying the rate for the whole region to the population at risk of each BHZ, represents the number of admissions for the condition under study that would have been observed in each BHZ under the hypothesis of constant rate across the whole Valencia region.

To assess the geographical variation in standardized hospitalization ratios, we used the Shared Component Model, which allows to analyze jointly several conditions by decomposing the spatial pattern of each one into two components: one shared by all conditions, and the other that is specific to each one. The SCM has as first-level assumption for the observed counts: O_ij_ ~ Poisson (μ_ij =_ e_ij_ρ_ij_), being ρ_ij_ the unknown relative risk for the BHZ *i* in the condition *j*. The second level stage assumes a common structure of risks using a random effect that is shared by the six PPH conditions plus random effects specific to each of them. This is achieved assuming log(μ_ij_) = log(e_ij_) + α_j_ + δ_j_φ_i_ + ϵ_ij_, where α_j_ values are the intercepts for each *j-th* PPH condition, ϵ_ij_ are the corresponding specific effects, and φ_i_ the random effect representing the shared component of the risk. The δ_j_ parameter is a scaling parameter that can be interpreted as a measure of the strength of the association between the shared term and the *j-th* PPH condition, which is comparable among PPH.

For the specific random effects ϵ_ij_, we assumed an exchangeable distribution, whereas for the common random effect φ_i_, we assessed two different specifications: an exchangeable distribution, and a conditional autoregressive distribution (CAR). The hyper-prior specifications used to carried out the Bayesian estimation procedure were: a *dflat* distribution for α_j_ and a normal distribution N(0,5.9) for log(δ_j_) [21,30]. For comparison purposes, we also fitted the so-called BYM model [29] for each condition, which has the same first-level assumption as the SCM models, but use independent random effects for each condition that take into account the spatial correlation through a CAR structure [31]. Model comparisons between the two competing shared component models, based on DIC statistics, [32] suggested the superiority of the exchangeable distribution (DIC(pD) = 8156.3(887.1)) over the CAR prior assumption (DIC(pD) = 8205.5(937.5)). The SCM exchangeable distribution model has also better DIC than the sum of the DIC values of the individual BYM models, with a difference of 61.5 points, showing a clear advantage of the joint over the individual modeling. Therefore, we selected the SCM exchangeable specification as a definitive SCM model.

All models were implemented in R, version 2.13.1, via the library R2WinBUGS (R Development Core team, 2007), which connects with the software WinBUGS [33]. The estimation procedure was carried out using Monte Carlo methods based on three Markov chains. A total of 49,500 iterations per chain were used, and after a burning period of 12,000 iterations, we kept every 75th for posterior inference. Convergence was determined using the Brooks and Gelman statistic and sequential and autocorrelation graphs. Scripts for SCM and BYM models are given in ‘Additional file 1’.

## Results

Considering the six PPH conditions all together, there were 39,970 hospital admissions in the Valencia Community between 2007 and 2009, ranging from 1 to 801 per BHZ. The person-years at risk were 13,131,836 (4,377,279 in annual average, ranging from 4,065 to 250,616 per BHZ). The highest admission rates (Table 1) corresponded to COPD (155 per 100,000 person-years [py]) and CHF (88.9 per 100,000 py), and the lowest to dehydration (3.9 per 100,000 py) and diabetes (11.3 per 100,000 py). Variability between Basic Health Zones was moderate for CHF (EB = 0.14), but high for the rest of conditions, especially for Dehydration (EB = 0.73), Angina (EB = 0.61) and Asthma (EB = 0.47). Correlation amongst the BHZ SHRs was high between COPD and CHF (r = 0.51), Asthma (r = 0.44) and Angina (0.34), but the remaining pair-wise combinations (except for CHF and Asthma; r = 0.33) showed low correlations (r < 0.30).

**Table 1:** PPH standardized rates by 100,000 person-years, variability among Basic Health Zones and correlation between PPH standardized hospitalization ratios of the different conditions; Valencia Community, 2007-2009

	**Summary data**		**Variability**		**Correlation (p-value) between standardized ratios**					
**PPH**	**n**	**Rates**	**EQ**_**5–95**_	**EB**	**Diab.**	**COPD**	**CHF**	**Dehyd.**	**Angina**	**Asthma**
Diabetes	1490	11.3	-	0.30	1.00	0.26 (<0.001)	0.16 (0.014)	-0.02 (0.805)	0.10 (0.107)	0.09 (0.159)
COPD	20357	155.0	6.12	0.26		1.00	0.51 (<0.001)	0.24 (<0.001)	0.34 (<0.001)	0.44 (<0.001)
CHF	11680	88.9	4.78	0.14			1.00	0.22 (0.001)	0.21 (0.001)	0.33 (<0.001)
Dehydration	512	3.9	-	0.73				1.00	0.23 (<0.001)	0.20 (0.002)
Angina	3141	23.9	34.24	0.61					1.00	0.22 (<0.001)
Asthma	2790	21.2	34.43	0.47						1.00

PPH: Potentially Preventable Hospitalizations; BHZ: Basic Health Zones; COPD: Chronic Obstructive Pulmonary Disease; CHF: Congestive Heart Failure. EQ: Extremal Quotient; EB: Empirical Bayes Statistics. EQ_5–95_ for Diabetes and Dehydration were not computable because BHZ with 0 cases.

According to the results of the Shared Component Model (Table 2), the estimates for the specific components vary greatly among PPH, CHF being the lowest (σ_specific_ = 0.079) and Dehydration the highest (σ_specific_ = 0.673). The estimate for the shared component was σ_shared_ = 0.121(95CI: 0.046-0.269). The proportion of variance for each PPH explained by the common patterns of the SCM was high for COPD (58.7%) and CHF (46.4%), and moderate for the rest (from 25.9 to 30.2%), which is also in agreement with the correlations among PPH Standardized Ratios shown in Table 1. In any case, credible intervals show a great imprecision. Comparing the strength of the association between each of the PPH-specific admissions risks patterns and the latent shared admission risk pattern, the factor loadings (δ) seems higher for Dehydration, COPD, Angina and Asthma than for Diabetes and CHF, although all 95% posterior credible intervals for the factor loadings include the unit and, therefore, the level of importance that the share component has on each PPH does not differ significantly among PPH (see ‘Additional file 2’ for a graphic illustration of the correlations between the spatial pattern relative risk for individual PPH and the spatial pattern relative risk shared by all conditions).

**Table 2:** Results of the shared component modelling

**PPH**	**σ (specific pattern variability)**	**δ (factor loadings)**	**% Variance explained by the shared component**
Diabetes	0.243 (0.102 - 0.377)	0.92 (0.46 - 1.68)	29.8 ( 5.7 - 70.6)
COPD	0.112 (0.040 - 0.211)	1.12 (0.77 - 1.68)	58.7 (22.0 - 84.7)
CHF	0.079 (0.035 - 0.117)	0.74 (0.47 - 1.47)	46.4 (22.3 - 75.2)
Dehydration	0.673 (0.249 - 1.111)	1.42 (0.80 - 2.52)	25.9 (5.3 - 73.1)
Angina	0.489 (0.116 - 0.697)	1.11 (0.62 - 2.82)	26.1 (4.1 - 83.4)
Asthma	0.353 (0.107 - 0.529)	1.09 (0.49 - 2.08)	30.2 (4.1 - 78.7)

PPH: Potentially Preventable Hospitalizations; COPD: Chronic Obstructive Pulmonary Disease; CHF: Congestive Heart Failure. The σ parameter describes the specific patterns for each condition of the Shared Component Model. The δ parameter is a measure of the strength of the association between the shared pattern for all 6 conditions and the specific pattern for each condition.

**Figure 1:**
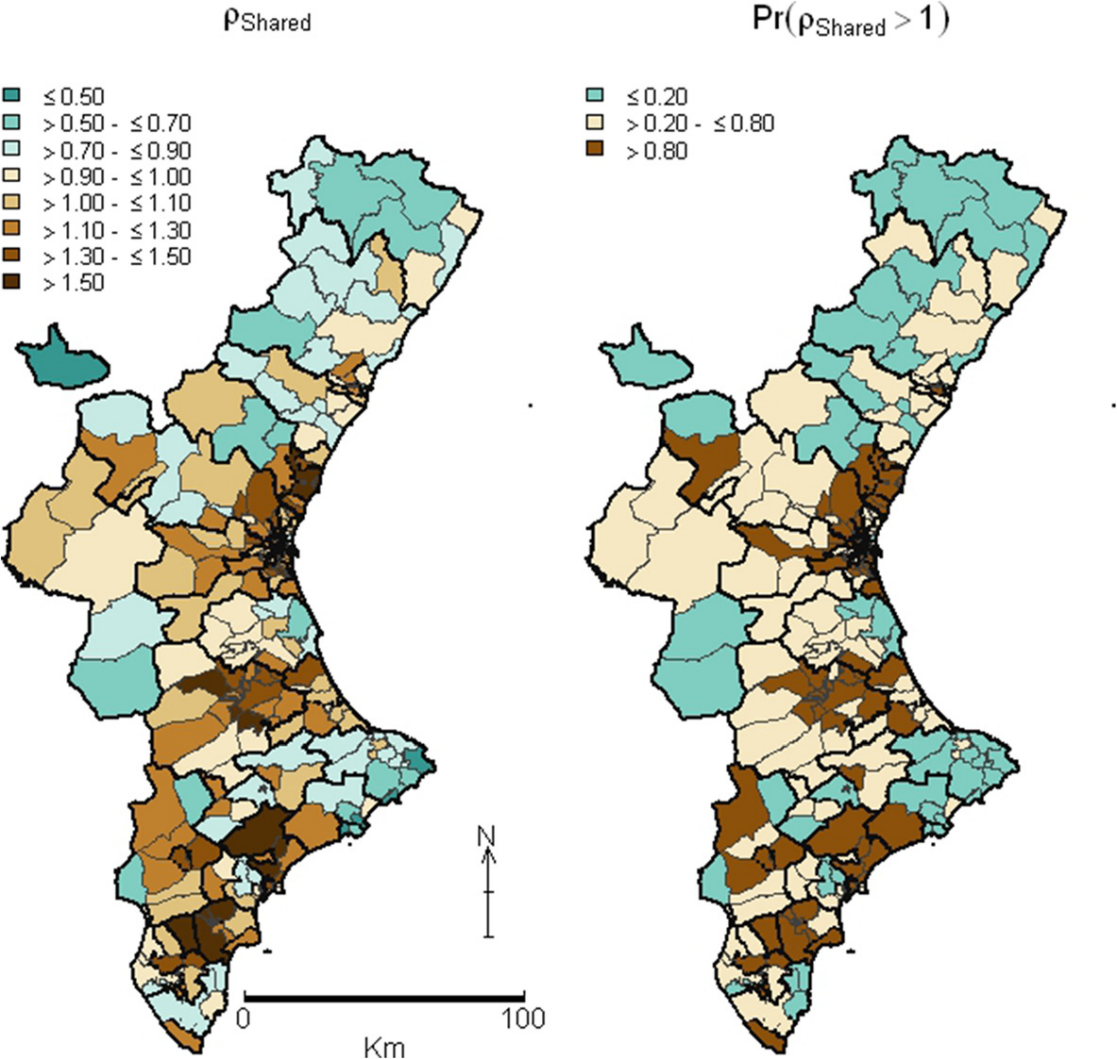
**Map of the pattern shared jointly by all six PPH conditions (left) and map with posterior probabilities that shared pattern were higher than1 (right).** Valencia Community, 2007–2009. Thin lines represent the boundaries between BHZ and thick lines the boundaries between Healthcare Areas. In the map of PPH shared pattern (left) colours represent relative risks regarding the jointly Valencia Community shared pattern of PPH admissions. In the map of posterior probability (right) dark-brown coloured territories are BHZ for which the probability of having a relative risk of PPH admissions (shared pattern) higher than 1 is >0.8, and green ones are BHZ for which this relative risk is below <0.2. PPH: Potentially Preventable Hospitalizations; BHZ: Basic Health Zones.

**Figure 2:**
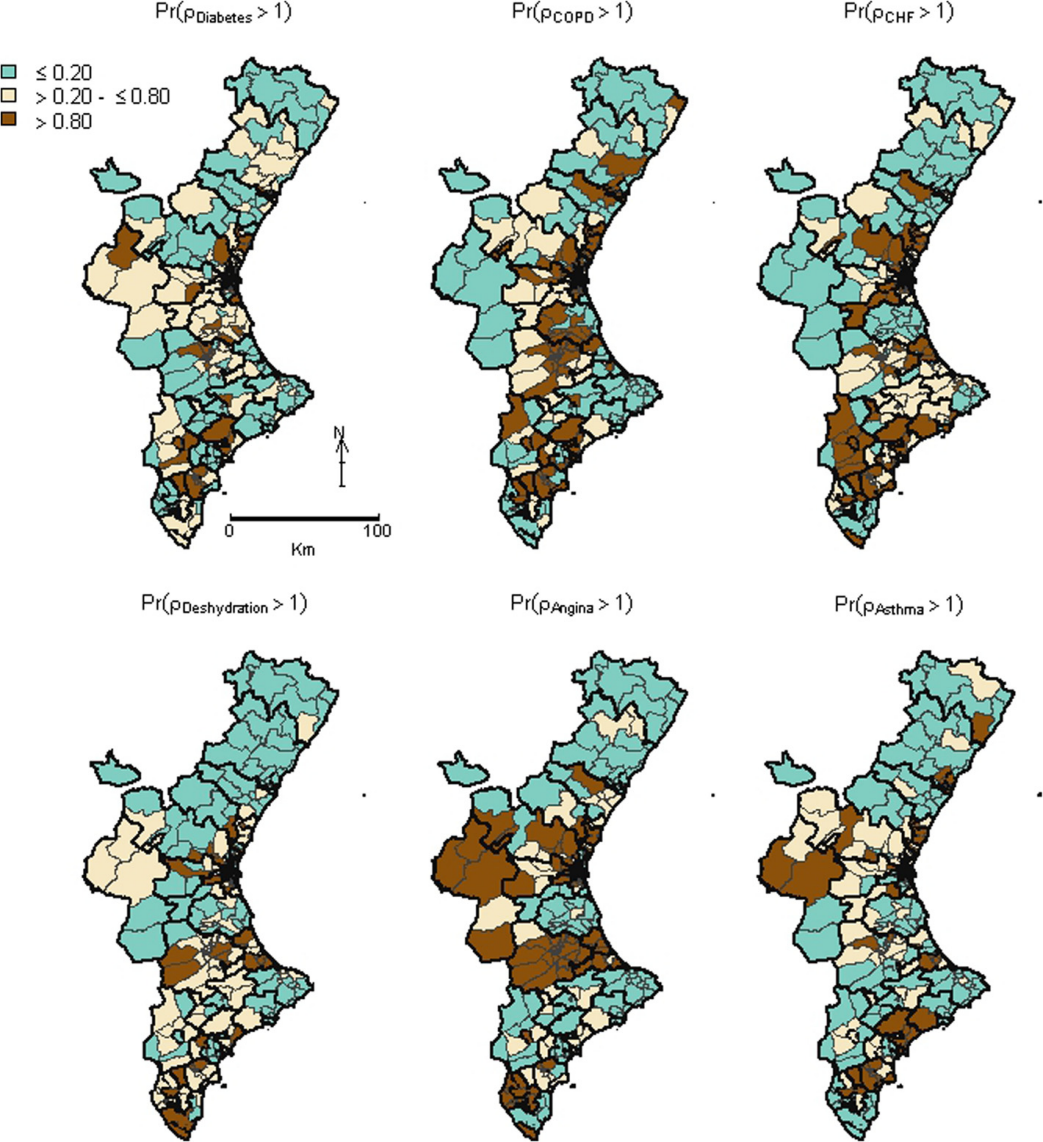
**Maps of posterior probabilities that the relative risk for the respective Potentially Preventable Hospitalization was higher than 1.** Dark-brown coloured territories are BHZ for which the probability of having a relative risk of PPH admissions higher than 1 for the respective condition is >0.8, and green ones are BHZ for which this relative risk is below <0.2. PPH: Potentially Preventable Hospitalizations; BHZ: Basic Health Zones.

**Figure 3:**
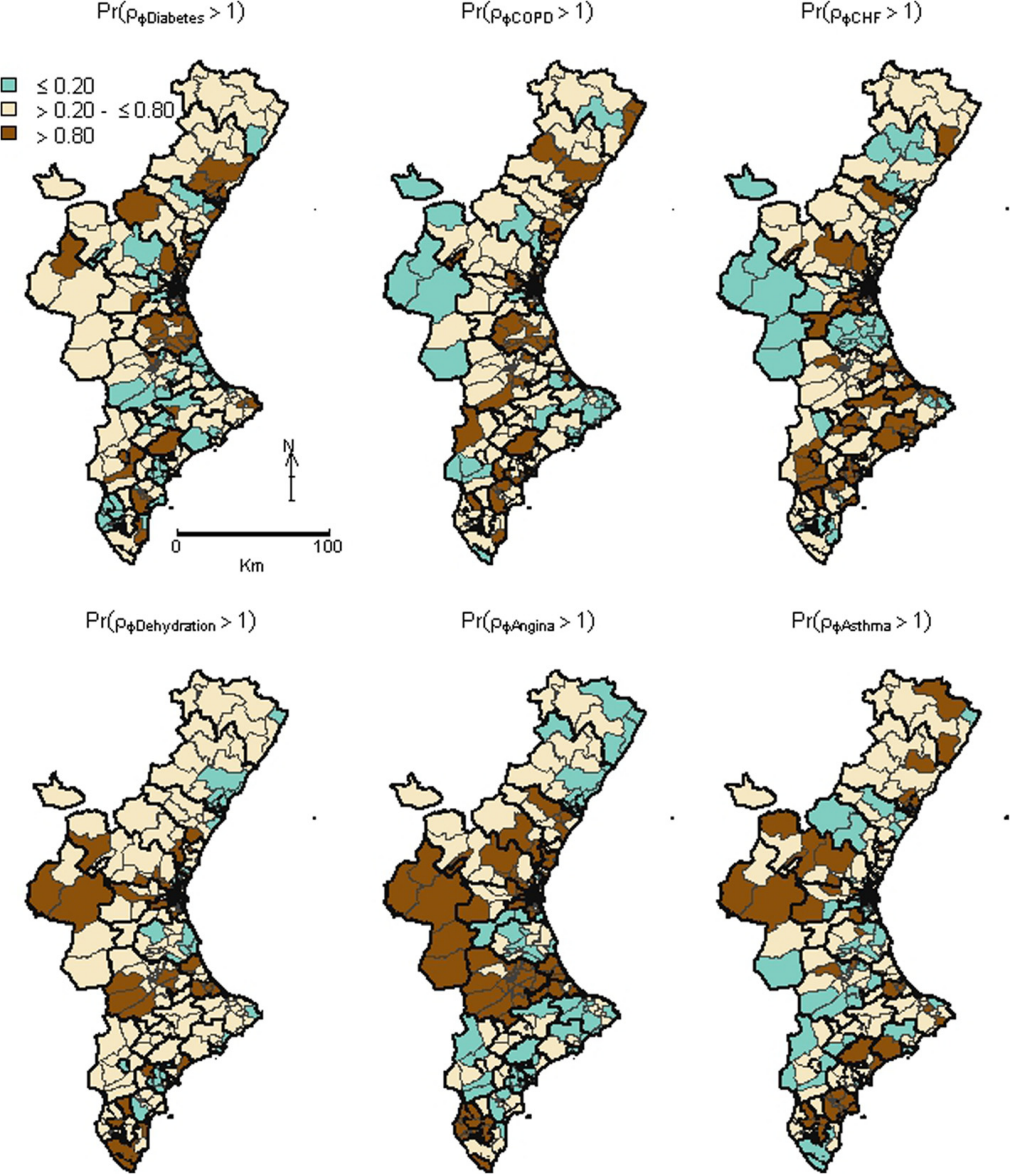
**Maps of posterior probabilities that the discrepant component (exp(ϵij)) regarding the shared pattern for the respective Potentially Preventable Hospitalization was higher than 1.** Dark-brown coloured territories are BHZ for which the probability of having a relative risk of PPH admissions higher than 1 for the respective discrepant component is >0.8, and green ones are BHZ for which this relative risk is below <0.2. PPH: Potentially Preventable Hospitalizations; BHZ: Basic Health Zones.

The shared component (term σ_Shared_ = exp(φ_i_)) is mapped in Figure 1, together with the map of the posterior probability that this shared component was above 1. The spatial pattern picks out three main clusters where the common pattern is particularly high, which coincides with three urban areas of the Valencia Region: the metropolitans area of the capital’s of the Valencia (in the middle) and Alicante (southern) provinces, and an area bordering these two provinces. In contrast, the whole province of Castellón (north) and one Healthcare Area in the southwest were the areas with lowest PPH admission risk. This spatial structure suggests a non-random pattern, which is confirmed by the Moran’s I estimate, which is 0.431 (p = 0.010).Figure 2 shows the maps for the posterior probabilities of the smoothed SHRs for each condition. Systematic similarities among maps are clearly depicted, such as the lower probability of higher risks in the northern third and the south-western side of the province. Nevertheless, high differences are also found, such as the strong spatial structure observed for Angina, or the high risk observed for Angina and Asthma in the west region, which has low risk for other PPH. Figure 3 plots the maps of the PPH-specific components, that is, the risk patterns that each PPH does not share with the common pattern. Maps are much smoother compared with the common pattern posterior maps, with many non-significant areas for most PPH, bearing out the important effect of the shared component. Nevertheless, some PPH such as Angina have a marked discrepant high-risk pattern, with significant high-risk areas in the middle-interior regions.

## Discussion

Our study shows, primarily, the possibility of modeling the geographical distribution of the risk of admission for several Potentially Preventable Hospitalizations concomitantly, providing the latent pattern shared by all the PPH conditions analyzed and quantifying how well each PPH is represented on it. The distribution of this common latent pattern captures about the 36% of the total variability, and may be used to evaluate healthcare systems performance, as it allows detecting areas with homogeneously low or high risk of PPH admissions that may suggest hypotheses about possible causes (and possible interventions) to reduce the volume of PPH. In the case studied (Valencia region), for example, the spatial structure of the PPH common patterns shows a non-random pattern, with local effects and rural–urban trends, practically defining the borders of some Healthcare Areas, which suggests that PPH may have an important relationship with specialist care common to all BHZ of the same Area. Although this hypothesis cannot be tested with the current design, as it does not explicitly consider the possible role of Healthcare Areas, it is important to highlight it, because it weakens the common belief that PPH are almost exclusively a problem of primary care, incorporating specialist care (without excluding hospitalization, emergency departments and some intermediate units such as hospital at home) to the definition of the problem, and shifting the attention to the coordination between the two levels of care.

The analysis of the single PPH divergence distribution may allow the identification of areas where more specific organizational factors could influence the rates of a particular PPH. This would be probable if some of these conditions were managed under shared, and divergent, care between primary and specialty care. In any case, the marked differences for some BHZ in specific PPH rates regarding the predominant pattern of their corresponding Healthcare Area suggest that in these cases the attention should be focused on the primary care teams responsible for these deviating BHZ.

A different approach for analyzing different PPH conjointly was proposed by a working group from AHRQ in 2006 [34] and included in later versions of the AHRQ PQIs [35]. These composite PQIs were planned to improve the statistical precision of the individual PQIs (increasing the numerator, even at the cost of losing internal homogeneity) allowing for greater discrimination in performance among areas and improved ability to identify differences and potential determining factors in performance. The final proposal included three composite indicators (acute, chronic and all PQIs) by summing the hospitalizations across different conditions and dividing by the population (the number of hospitalizations is used as the “weight” for combining the different entities). In the latent variable method proposed in the present study, the shared pattern could also be considered as a composite measure, being the most prevalent conditions (COPD, CHF) more represented. Additionally, the SCM method allows the identification of causes of avoidable hospitalizations whose behaviour is significantly discrepant regarding this “composite indicator”, opening the possibility to new hypotheses, but at the expense of a more complex analysis than the composite AHRQ PQI measures.

Our study has some strengths and some limitations. Among the strengths it should be noted the organizational characteristics of the VHA, with hospital and primary care service areas administratively defined, facilitating the analysis of small areas and their interpretation in terms of healthcare providers and levels of care. Also, the use of a three-year period, allows having enough statistical power for low incidence PPH, improving the stability of the estimates. From a methodological point of view, besides providing separate maps for the shared and specific components of the risk surface, [19] and advantage of this approach is the ability of the model to pool data and borrow strength among multivariate health outcomes and across neighbouring geographical areas for more reliable small area risks prediction and inference [20]. Among the limitations, first, hospitalizations in private hospitals were not included, and even though we excluded populations with private insurance, we could not exclude patients with a double insurance. The significance and direction of this bias is difficult to estimate, but given the expected better socioeconomic situation of people with additional private insurance, and that Valencia private hospitals are relatively more specialized in elective surgery and deliveries than in chronic conditions, we think it may not be important. Nor were there included hospitalizations occurred outside the VHA because of inaccessibility of data, but we think this could only affect to a relatively small number of admissions on the causes under study. Second, hospital admission rates, as some authors have pointed out, [36,37] even standardized by age and sex, do not fully account for the differences in disease prevalence between areas, or in the distribution of particularly vulnerable subpopulations, this last an aspect that has already been proved to be associated with PPH in many regions of Europe [38,39] and USA [40]. Third, the quality of the information of the Minimum Basic Hospital Discharge Dataset has not been exhaustively analysed in the literature, and may not be optimal for research. Nevertheless, after having undergone a standardization procedure, it is considered one of the administrative information datasets with greatest scope and usefulness for research [41]. And last, the PPH definitions used were recently validated in Spain, [24,25] an aspect which increases its internal validity, however this same consideration limits the contrast of our results with studies that used other lists (including other lists in Spain).

## Conclusions

In summary, this paper shows that the shared component modelling offers useful information when analyzing PPH at community level. It provides a global picture of the geographical pattern shared by different PPH, improving our ability to evaluate healthcare systems performance, and at the same time it depicts the particularities of each PPH, an aspect that could be connected with particular organizational factors affecting specific territories.

## Competing interests

The authors declare that they have no competing interests.

## Authors’ contributions

BI and JL had full access to all of the data in the study and take responsibility for the integrity of the data and the accuracy of the data analysis. BI, JL, EBD, SGA, SMF and SP, were responsible for the study concept, design and data acquisition. BI and JL carried out the data preparation and carried out the statistical analysis and BI, JL, SP and EBD drafted the manuscript. All authors participated in the analysis and interpretation of data, critical revision of the manuscript for important intellectual content, and all approved the final version submitted for publication.

## Pre-publication history

The pre-publication history for this paper can be accessed here:

http://www.biomedcentral.com/1471-2288/14/74/prepub

## Supplementary Material

Additional file 1 Script for SCM and BYM models. Click here for file

Additional file 2 Correlations between the SCM relative risks for individual PPH and the SCM estimated relative risk shared by all conditions. Click here for file
