# Supplementary material for: Joint spatial modeling to identify shared patterns among chronic related potentially preventable hospitalizations
Source: BMC Med Res Methodol. 2014 Jun 4;14:74. doi: 10.1186/1471-2288-14-74 (PMC4053553; doi:10.1186/1471-2288-14-74)
Supplement: Additional file 1 — Script for SCM and BYM models. [file 1471-2288-14-74-S1.docx]

**SCRIPT FOR MODEL SCM WITH EXCHANGEABLE SHARED COMPONENT**

model

{

for (i in 1 : N_zbs) {

for (j in 1:N_dis) {

# Poisson likelihoods

O[i,j] ~ dpois(mu[i,j])

RRrho[i,j]<-mu[i,j]/E[i,j]

log(mu[i,j]) <- log(E[i,j])+ alpha[j] + epsilon[i,j] +v[i,j]

}

}

for(i in 1:N_zbs) {

# Define log relative risk in terms of disease-specific (epsilon) and shared (phi) random effects, with weights (delta)

v[i,1] <- delta[1]*phi[i]

v[i,2] <- delta[2]*phi[i]

v[i,3] <- delta[3]*phi[i]

v[i,4] <- delta[4]*phi[i]

v[i,5] <- delta[5]*phi[i]

v[i,6] <- delta[6]*phi[i] }

# prior model for the shared random effects

for (i in 1:N_zbs){

phi[i] ~ dnorm(0, tau.spatial)

}

# Multivariate Normal distribution for the unstructured components

for (i in 1:N_zbs) {

epsilon[i,1:N_dis] ~ dmnorm(mean[1:N_dis], P[1:N_dis, 1:N_dis])

}

# Priors for the intercepts, fixed effects, relative weight and variances

for (j in 1:N_dis) {

alpha[j] ~ dflat()

alphaRR[j]<-exp(alpha[j])

}

for (j in 1:N_dis) {

logdelta[j] ~ dnorm(0, 5.9)

delta[j] <- exp(logdelta[j])

}

tau.spatial ~ dgamma(0.5, 0.0005)

sigma.spatial <-1/tau.spatial

for (j in 1:N_dis) {mean[j] <-0.0 }

P[1:N_dis,1:N_dis] ~ dwish(Q[1:N_dis, 1:N_dis],N_dis)

for (i in 1:N_dis) {

for (j in 1:N_dis) {

Q[i,j]<-0.01*equals(i,j)}}

# relative risks and other summary quantities

for (i in 1 : N_zbs) {

sharedRR[i]<- exp(phi[i]) # overall RR of shared in zbs i

prob.sharedRR[i]<-step(sharedRR[i]-1)

}

for (i in 1 : N_zbs) {

for (j in 1:N_dis) {

especificRR[i,j]<-exp(epsilon[i,j]) # residual RR of dis[j] in zbs i not explained by shared component

}

}

for (j in 1:N_dis) {

var.especific[j]<-sd(epsilon[,j])*sd(epsilon[,j]) # empirical variance of disease specific effects

var.shared[j]<-pow(delta[j],2)*sd(phi[])*sd(phi[]) # empirical

frac.shared[j] <-(pow(delta[j],2)*sd(phi[])*sd(phi[])) / (var.shared[j] + (sd(epsilon[,j])*sd(epsilon[,j])))

}

}

**SCRIPT FOR MODEL SCM WITH CAR SHARED COMPONENT**

model

{

for (i in 1 : N_zbs) {

for (j in 1:N_dis) {

# Poisson likelihoods

O[i,j] ~ dpois(mu[i,j])

RRrho[i,j]<-mu[i,j]/E[i,j]

log(mu[i,j]) <- log(E[i,j])+ alpha[j] + epsilon[i,j] +v[i,j]

}

}

for(i in 1:N_zbs) {

# Define log relative risk in terms of disease-specific (epsilon) and shared (phi) random effects, with weights (delta)

v[i,1] <- delta[1]*phi[i]

v[i,2] <- delta[2]*phi[i]

v[i,3] <- delta[3]*phi[i]

v[i,4] <- delta[4]*phi[i]

v[i,5] <- delta[5]*phi[i]

v[i,6] <- delta[6]*phi[i] }

# Spatial prior model for the shared random effects

phi[1:N_zbs] ~ car.normal(adj[], weights[], num[], tau.spatial)

# Weights for the spatial adjacency matrix

for(k in 1:sumNumNeigh) {

weights[k] <- 1

}

# Multivariate Normal distribution for the unstructured components

for (i in 1:N_zbs) {

epsilon[i,1:N_dis] ~ dmnorm(mean[1:N_dis], P[1:N_dis, 1:N_dis])

}

# Priors for the intercepts, fixed effects, relative weight and variances

for (j in 1:N_dis) {

alpha[j] ~ dflat()

alphaRR[j]<-exp(alpha[j])

}

for (j in 1:N_dis) {

logdelta[j] ~ dnorm(0, 5.9)

delta[j] <- exp(logdelta[j])

}

tau.spatial ~ dgamma(0.5, 0.0005)

sigma.spatial <-1/tau.spatial

for (j in 1:N_dis) {mean[j] <-0.0 }

P[1:N_dis,1:N_dis] ~ dwish(Q[1:N_dis, 1:N_dis],N_dis)

for (i in 1:N_dis) {

for (j in 1:N_dis) {

Q[i,j]<-0.01*equals(i,j)}}

# relative risks and other summary quantities

for (i in 1 : N_zbs) {

sharedRR[i]<- exp(phi[i]) # overall RR of shared in zbs i

prob.sharedRR[i]<-step(sharedRR[i]-1)

}

for (i in 1 : N_zbs) {

for (j in 1:N_dis) {

especificRR[i,j]<-exp(epsilon[i,j]) # residual RR of dis[j] in zbs i not explained by shared component

}

}

for (j in 1:N_dis) {

var.especific[j]<-sd(epsilon[,j])*sd(epsilon[,j]) # empirical variance of disease specific effects

var.shared[j]<-pow(delta[j],2)*sd(phi[])*sd(phi[]) # empirical

frac.shared[j] <-(pow(delta[j],2)*sd(phi[])*sd(phi[])) / (var.shared[j] + (sd(epsilon[,j])*sd(epsilon[,j])))

}

}

**SCRIPT FOR MODEL BYM FOR EACH CONDITION INDEPENDENTLY**

model

{

for (i in 1 : N_zbs) {

O[i] ~ dpois(mu[i])

log(mu[i]) <- log(E[i]) + alpha+ b[i] + h[i]

RR[i]<-exp(b[i]+h[i])

h[i]~dnorm(0, tau.h)

PRP[i]<-step(RR[i]-1)

}

# CAR prior distribution for random effects:

b[1:N_zbs] ~ car.normal(adj[], weights[], num[], tau.b)

for(k in 1:sumNumNeigh) {

weights[k] <- 1

}

#other priors

alpha ~ dnorm(0.0, 1.0E-5)

sigma.h ~ dnorm(0,0.1)I(0, )

tau.h<- 1/(sigma.h*sigma.h)

sigma.h2<-sigma.h*sigma.h

sigma.b ~ dnorm(0,0.1)I(0, )

tau.b<- 1/(sigma.b*sigma.b)

sigma.b2<-sigma.b*sigma.b

sigma.b2marginal<-sd(b[])*sd(b[])

frac.spatial<-sigma.b2marginal/(sigma.b2marginal+sigma.h2)

}
