# Supplementary material for: Joint spatial modeling to identify shared patterns among chronic related potentially preventable hospitalizations
Source: BMC Med Res Methodol. 2014 Jun 4;14:74. doi: 10.1186/1471-2288-14-74 (PMC4053553; doi:10.1186/1471-2288-14-74)
Supplement: Additional file 2 — Correlations between the SCM relative risks for individual PPH and the SCM estimated relative risk shared by all conditions. [file 1471-2288-14-74-S2.doc]

The *figure* illustrates graphically the correlation between the estimated relative risks for each PPH vs. the relative risk of the shared pattern for all six PPH. For COPD and CHF, points are closer to the regression line (higher percent of variance explained) than for other conditions, while the PPH with greater slope (higher importance of the shared component) were Dehydration, COPD and Angina.

| Correlations between the SCM estimated spatial pattern relative risk for individual PPH (y-axis) and the SCM estimated spatial pattern relative risk shared by all conditions. |
| --- |
| 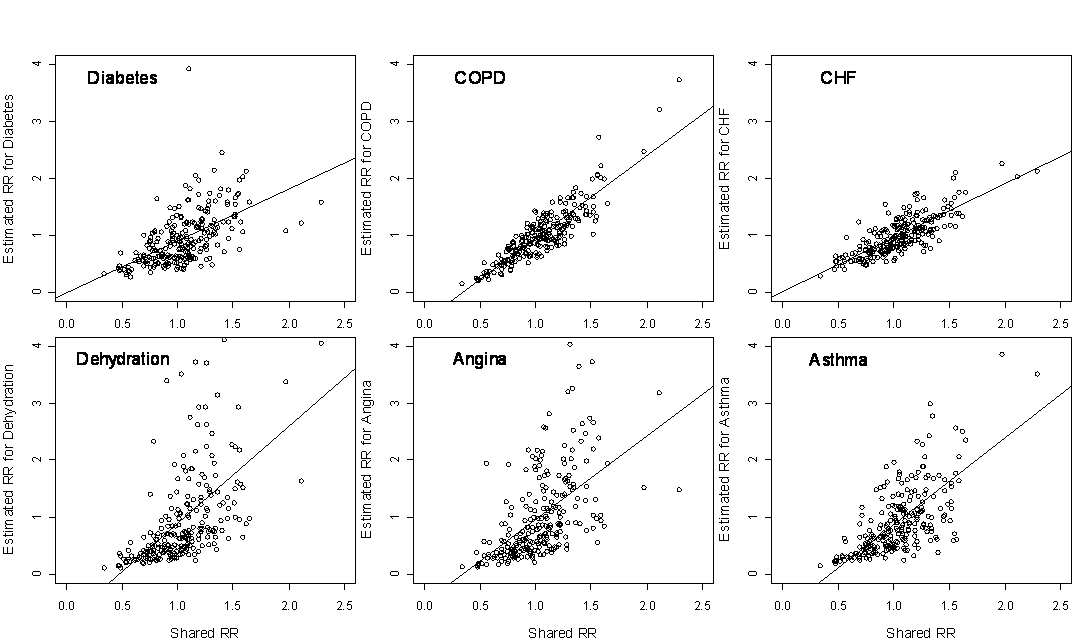 |
| SCM: Shared Component Model; PPH: Potentially Preventable Hospitalizations. COPD: Chronic Obstructive Pulmonary Disease; CHF: Congestive Heart Failure. |
